# Observation of the reversed Cherenkov radiation

**DOI:** 10.1038/ncomms14901

**Published:** 2017-03-23

**Authors:** Zhaoyun Duan, Xianfeng Tang, Zhanliang Wang, Yabin Zhang, Xiaodong Chen, Min Chen, Yubin Gong

**Affiliations:** 1 National Key Laboratory of Science and Technology on Vacuum Electronics in Chengdu, Institute of High Energy Electronics, School of Physical Electronics, University of Electronic Science and Technology of China, No. 4, Section 2, North Jianshe Road, Chengdu 610054, China; 2 School of Electronic Engineering and Computer Science, Queen Mary, University of London, London E1 4NS, UK; 3 Department of Physics, Massachusetts Institute of Technology, 77 Massachusetts Avenue, Cambridge, Massachusetts 02139, USA

## Abstract

Reversed Cherenkov radiation is the exotic electromagnetic radiation that is emitted in the opposite direction of moving charged particles in a left-handed material. Reversed Cherenkov radiation has not previously been observed, mainly due to the absence of both suitable all-metal left-handed materials for beam transport and suitable couplers for extracting the reversed Cherenkov radiation signal. In this paper, we develop an all-metal metamaterial, consisting of a square waveguide loaded with complementary electric split ring resonators. We demonstrate that this metamaterial exhibits a left-handed behaviour, and we directly observe the Cherenkov radiation emitted predominantly near the opposite direction to the movement of a single sheet electron beam bunch in the experiment. These observations confirm the reversed behaviour of Cherenkov radiation. The reversed Cherenkov radiation has many possible applications, such as novel vacuum electronic devices, particle detectors, accelerators and new types of plasmonic couplers.

## Abstract (web summary)

Reversed Cherenkov radiation has not been observed due to the absence of suitable all-metal left-handed materials for beam transport and suitable couplers for extracting the signal. Here, Duan *et al*. develop a metamaterial to observe reversed Cherenkov radiation using real charged particles.

Charged particles travelling through a dielectric medium with a speed greater than the phase velocity of an electromagnetic wave in that medium emit Cherenkov radiation. In conventional materials Cherenkov radiation propagates in the same direction as the particles, termed forward Cherenkov radiation. Forward Cherenkov radiation was experimentally discovered by Čerenkov in 1934 (ref. 1) and theoretically explained by Tamm and Frank in 1937 (ref. 2). Forward Cherenkov radiation is widely used in particle detectors, accelerators, high-power vacuum electronic devices, space science, and so on. For example, it was used to identify charged particles with different masses, but the same momentum, leading to the famous discoveries of the anti-proton^3^ and the J-particle^4^.

Reversed Cherenkov radiation (RCR) refers to the exotic electromagnetic radiation that is emitted in the opposite direction of moving charged particles in a left-handed material (LHM), theoretically predicted by Veselago^5^. Veselago also predicted that LHMs would exhibit a range of novel phenomena except for the RCR, such as a negative refractive index and a reversed Doppler effect^5^. The first LHMs were extensively studied in the 1990s (refs 6−8, 7, 8), with experimental observations of the negative refractive index in 2001 (ref. 9) and the reversed Doppler effect in a one-dimensional metamaterial in 2003 (ref. 10). These LHMs consisted of periodic arrays of subwavelength metal/dielectric geometric structures^9^, where the arrays can be described as an effective medium. Recent advances in metamaterial research have led to many potential applications, such as imaging^11^, cloaking^12^ and terahertz devices^13^.

Since 2003 numerous researchers have attempted to measure RCR in LHMs^14^,^15^,^16^,^17^,^18^,^19^,^20^,^21^,^22^,^23^,^24^,^25^, although none has been successful. For example, Argonne National Laboratory made an LHM inside a rectangular waveguide, but failed to observe RCR partly due to the coupler problems^24^, and an attempt made by Los Alamos National Laboratory did not succeed in realizing an LHM^25^. Therefore, several researchers attempted to do indirect experiments, including an analogue to RCR^26^, a phased dipole array to mimic moving particles in the frequencies ranging from 8.1 to 9.5 GHz (refs 27, 28), and similar work in the light band^29^,^30^. It should be noted that all the above LHMs used dielectric substrates, which in the vacuum environment required for charged particle transport would outgas, making these LHMs unsuitable for use.

Here, we show the left-handed property of our constructed all-metal metamaterial using the effective media model and dispersion relation. In the experiments, we use real charged particles from a single sheet electron beam (SEB) bunch to observe the RCR in the LHM. The physical mechanism of generating the novel RCR using real charged particles in the LHM is given. These findings shed light on the potential applications.

## Results

### Effective constitutive parameters

**Figure 1:**
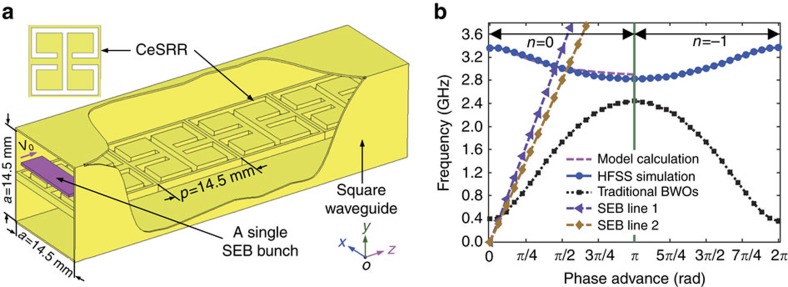
Constructed structure and associated dispersion. (**a**) Schematic diagram of the constructed structure interacting with a single SEB bunch travelling along the +*z* direction. (**b**) Dispersion curves characterized by frequency versus phase advance *φ* (*φ*=*β*_0_*p*) of the propagating wave, where *β*_0_ is the phase constant. The dispersion curve using the model calculation can be found in ref. 31. The HFSS simulation is based on the eigenmode solver. The SEB line is determined by 2*πf*=*β*_0_*v*_0_, where *f* is the frequency and *v*_0_ is the initial speed of the single SEB bunch, which is determined by *V*=(*γ*−1)*mc*^2^/*e*. Here *V=*160 kV and 99.7 kV are the beam voltages, which correspond to SEB lines 1 and 2, respectively; *γ*, *c*, *e*, and *m* are the relativistic factor, speed of light in vacuum, elementary electric charge and rest mass of electron, respectively. The black dotted line represents a schematic dispersion for a corrugated slow-wave structure.

In our RCR experiment, to avoid dielectric breakdown and to maintain a high vacuum, we constructed an all-metal LHM structure^31^, where the TM-dominant mode can interact with a single SEB bunch. As shown in Fig. 1a, the constructed structure consists of a square waveguide loaded with a layer of complementary electric split ring resonators (CeSRRs), with the period *p*=14.5 mm. Here the hollow square waveguide (called SW1), with a cross section of *a* × *a* (here *a*=14.5 mm), operates below the cutoff frequency for the TM_11_ mode, compared to normal waveguide. This shows that the period *p* equals the side length of the cross section of the square waveguide^32^. As the size of the CeSRR unit cell and the cross section of the waveguide are much smaller (∼*λ*/7) than the free-space wavelength (*λ*) at the operating frequencies, the CeSRR-loaded waveguide can be considered as the effective media. As presented in ref. 31, the effective permittivity tensor of the constructed structure is determined by the S-parameter retrieval method based on the HFSS simulations on a single CeSRR (Supplementary Fig. 1 and Supplementary Note 1) and it can be written as


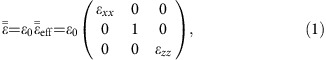


where *ɛ*_0_ is the permittivity in vacuum, and *ɛ*_*xx*_ and *ɛ*_*zz*_ are the effective permittivity parameters^31^. A hollow metallic waveguide operating at microwaves can be regarded as a one-dimensional lossless magnetic plasma with respect to the TM_11_ mode propagation along the axial direction. As a result, the effective permeability *μ*=*μ*_0_*μ*_eff_ can be predicted by the Drude model^32^, ^33^, ^34^, ^35^, ^36^, where *μ*_0_ and *μ*_eff_ are the permeability in vacuum and the relative permeability, respectively (Supplementary Fig. 1 and Supplementary Note 1). Note that for simplicity, only the real parts of the effective constitutive parameters are taken into account. The CeSRR layer forms an effective medium with negative electric response in the *x* direction, indicating that *ɛ*_xx_ is negative in a narrow frequency band^31^. The hollow square waveguide, operating below the cut-off frequency for the TM_11_ mode, can be considered as an effective medium with negative magnetic response, indicating that *μ*_eff_ is negative in the same frequency band as *ɛ*_*xx*_. This means our CeSRR-loaded waveguide can be considered as an LHM.

### Left-handed behaviour derived from dispersion

For further physical description of the effective media model, we use the eigenmode solver in HFSS to simulate the CeSRR-loaded waveguide and then obtain the dispersion relation. The dispersion curve, plotted in Fig. 1b, shows that the *n*=0 space harmonic of the fundamental mode exhibits a left-handed behaviour or backward dispersion, where *n* is the space harmonic index^37^. This is because the left-handed behaviour or backward dispersion of the LHM is established by the phase velocity and the group velocity propagating in opposite directions, which is the pre-condition for the onset of RCR. The good agreement between dispersion curves obtained from the simulation and the model calculation^31^ further demonstrates that our constructed structure can be considered as an LHM (Supplementary Fig. 2a and Supplementary Note 2). For comparison, the schematic dispersion curve for a corrugated slow-wave structure employed in a traditional backward-wave oscillator (BWO) is also plotted in Fig. 1b (black dotted line). This shows that for the traditional BWO the *n*=0 space harmonic of the fundamental mode exhibits a forward dispersion, while the *n*=−1 space harmonic presents a backward dispersion^38^.

For our LHM, the *n*=0 space harmonic of the fundamental mode is the TM-dominant wave (Supplementary Fig. 3), which demonstrates the validness of the assumption (the waveguide acts as the magnetic plasma). In addition, the field distributions in the real LHM (with effective constitutive parameters) are similar to those in the realistic structure (Fig. 1a). This is a great supporting argument for the effective media model presented here.

From Fig. 1b, the intersection point between the dispersion curve and the SEB line gives us the synchronous condition between the SEB bunch and the excited electromagnetic waves. The synchronous condition requires that the initial speed of the SEB bunch is slightly larger than the phase velocity of the coherent electromagnetic waves. The RCR frequency can be approximately obtained from the synchronous condition, and accurately predicted by the particle-in-cell (PIC) simulations, which we then confirm in the RCR experiment described below.

Based on the above dispersion curve from the HFSS simulation, we use post-processing in HFSS to calculate the interaction impedance (Supplementary Fig. 2b and Supplementary Note 2), an important parameter for describing the high-frequency characteristics of the LHM presented here. It is found that the large improvement of the longitudinal electric field intensity in the LHM leads to a much higher interaction impedance compared with conventional slow-wave structures. This result implies that an LHM is not only suitable for observing RCR^31^ but can also be used to develop novel vacuum electronic devices with higher electronic efficiency^39^ relative to conventional BWOs.

### Left-handed behaviour obtained from the phase spectra

**Figure 2:**
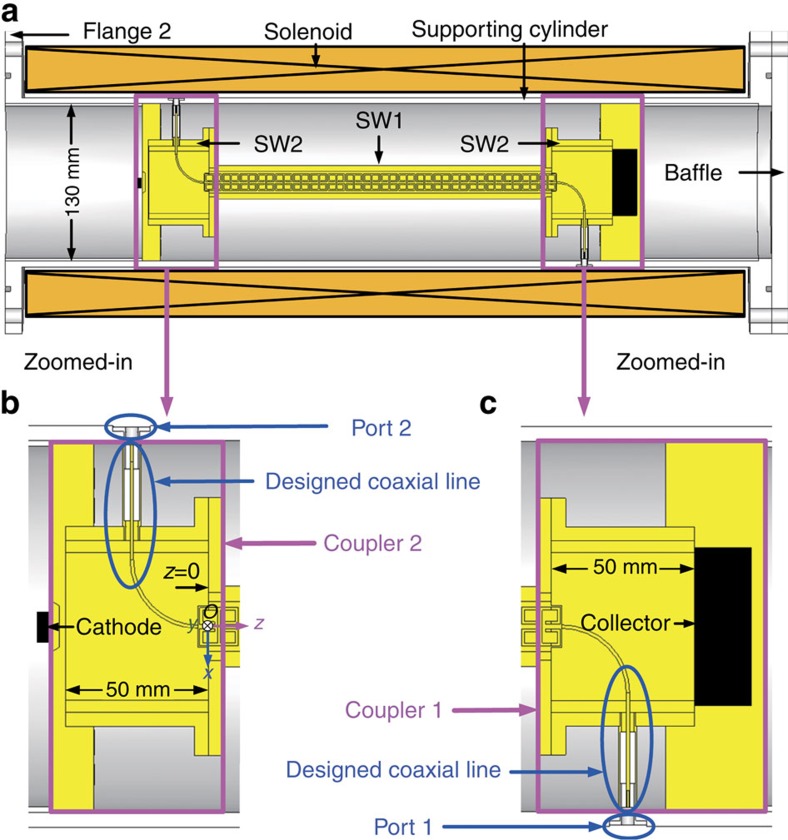
Schematic of the device under test for RCR. (**a**) The CeSRR layer with two couplers is located in the centre of the SW1 that is covered by a solenoid. (**b**) The corresponding zoomed-in section shows coupler 2. (**c**) The corresponding zoomed-in section shows coupler 1.

In addition, we use transmission-amplitude/phase spectra measurements to demonstrate the passband and left-handed behaviour of our constructed structure. Here we have constructed two couplers (coupler 1 and coupler 2) to detect RCR, as shown in Fig. 2. To connect the system to a standard coaxial line, we constructed a specially designed coaxial line, where the inner conductor includes a straight section and a *π*/2-radians arc section. Thus, each coupler consists of a square waveguide (called SW2) and the designed coaxial line. We verified the performance of our couplers via simulations and experiments, the results of which are presented below.

**Figure 3:**
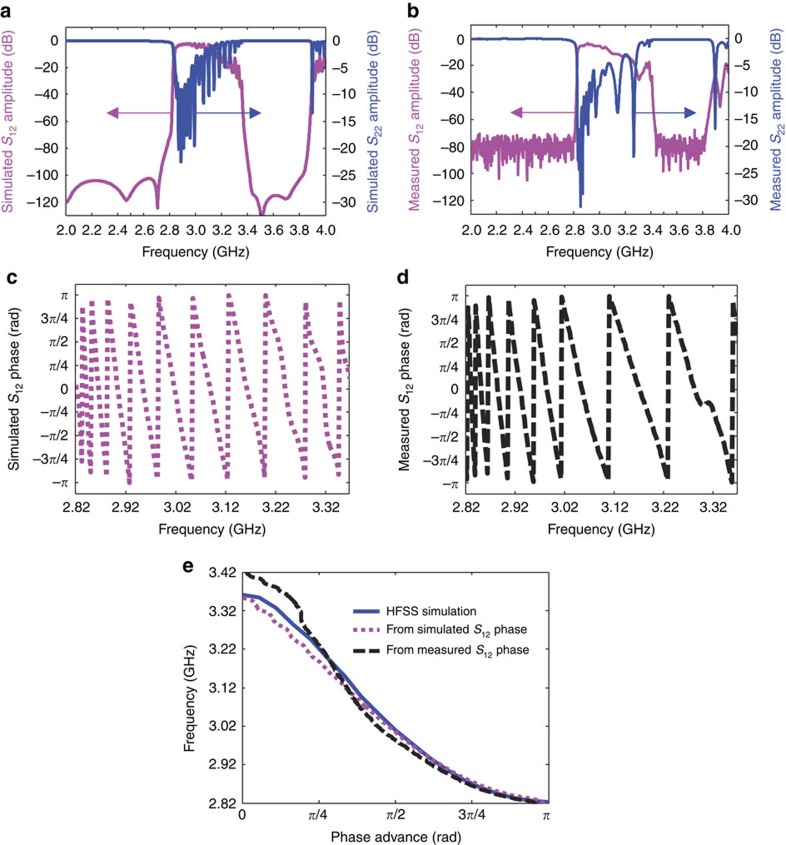
Transmission property of electromagnetic waves in the LHM. (**a**) Simulated amplitudes of transmission coefficient (*S*_12_) (magenta solid line) and reflection coefficient (*S*_22_) (blue dashed line). (**b**) Measured amplitudes of transmission coefficient (*S*_12_) (magenta solid line) and reflection coefficient (*S*_22_) (blue dashed line). (**c**) Simulated S_12_ phases versus frequency. (**d**) Measured *S*_12_ phases versus frequency. (**e**) Comparison of dispersion curves from the HFSS simulation (blue solid line, also shown in Fig. 1b), simulated *S*_12_ phase (magenta dotted line) and measured *S*_12_ phase (black dashed line).

The simulations were performed using the frequency domain solver in CST Microwave Studio to compute transmission and reflection coefficients (S-parameters) across a realistic model (Fig. 2a). Comparative experiments using an Agilent N5230A PNA-L Network Analyzer to measure the S-parameters across the two couplers, without the SEB bunch, were also conducted (Supplementary Figs 4 and 5 and Supplementary Note 3). The amplitudes for the S-parameters are shown in Fig. 3a,b. We see that the simulated amplitudes are consistent with the measured values and there is a passband from 2.83 to 3.05 GHz, where electromagnetic waves can efficiently propagate (high transmission, low reflection). This would allow an excited RCR signal, in the passband, to be efficiently extracted from port 2 for detection.

Using the simulated and measured *S*_12_ phases (Fig. 3c,d) the dispersion curves can be derived, as shown in Fig. 3e. We find that the dispersion curves agree well with the HFSS simulation conducted earlier. As a result, the *S*_12_ phases also verify the left-handed behaviour in the frequency range of 2.83–3.05 GHz from simulations and experiments (Supplementary Fig. 6 and Supplementary Note 3).

### RCR simulations using the PIC solver

**Figure 4:**
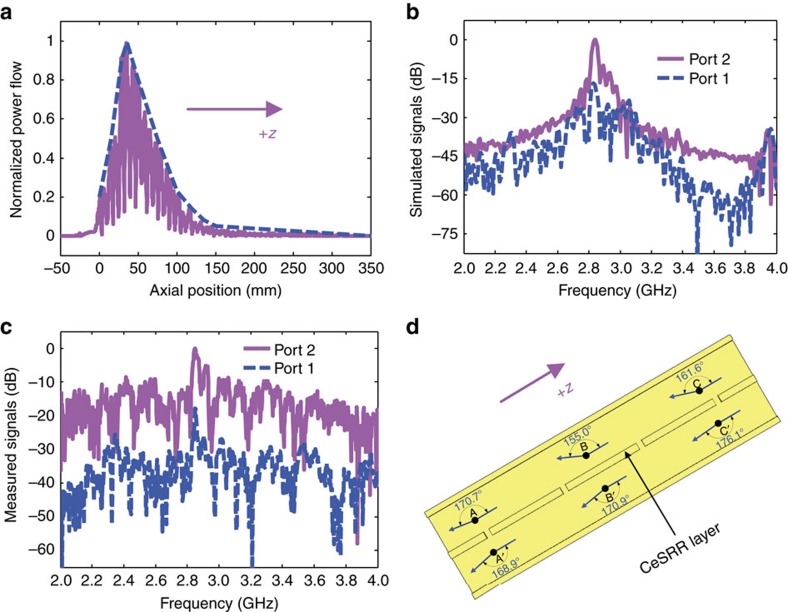
RCR physical features. (**a**) Simulated *z*-component amplitudes of the power flow (*x*=0 mm, *y*=2.1 mm) normalized to its maximum value (magenta curve) versus the axial position within the LHM area at 20 ns. The central positions of the coaxial lines for ports 2 and 1 are located at *z*=−26.8 mm and *z*=309.6 mm, respectively. The smooth blue curve represents the RCR signal envelope. (**b**) Simulated power spectral densities of the RCR and its reflection signals at ports 2 and 1, respectively, which are normalized to the maximum value of the simulated RCR signal. (**c**) Measured power spectral densities of the RCR and its reflection signals at ports 2 and 1, respectively, which are normalized to the maximum value of the measured RCR signal. (**d**) Simulated Cherenkov radiation angles at different positions A (0, 3.2, 10 mm), B (0, 3.2, 25 mm), C (0, 3.2, 40 mm), A′ (0, −3.2, 10 mm), B′ (0, −3.2, 25 mm) and C′ (0, −3.2, 40 mm) at 20 ns.

To investigate RCR behaviour numerically we used the PIC solver in CST Particle Studio to simulate the interaction of an electron bunch in our geometry with the excited RCR (Supplementary Fig. 7 and Supplementary Note 4). In the simulations, a single 160 kV, 1.55 kA, 20 ns pulse SEB with a cross section of 12 mm × 2 mm is used, and the two couplers are utilized as outputs for any generated signals. Because the space and time are dependent in the beam-wave interaction, during the transient state of the RCR, as time (*t*) increases, from *t*=0 ns corresponding to the axial position *z*=282.8 mm, the RCR is first excited and gradually amplified along the opposite direction of the SEB bunch's movement, which means *z* decreases with it. The transient state ends at about 20 ns, which means the RCR starts in the steady state. Therefore, the *z*-component amplitudes of the power flow normalized to its maximum value versus the axial position at 20 ns are shown in Fig. 4a. The peak at *z*=35 mm corresponds to the maximum RCR intensity, which can be efficiently coupled out of the system through coupler 2. In addition, the simulated microwave signals at ports 2 and 1 are plotted in Fig. 4b. We see that at 2.829 GHz the power at port 1 is 16.9 dB lower than the 2.836 GHz RCR at port 2. Note that the signal at port 1 is reflected from port 2 due to the imperfect impedance match at coupler 2.

### RCR experiments using the LHM and couplers

After the RCR simulations presented above, we carried out the RCR experiments (Supplementary Fig. 8 and Supplementary Note 4). A Tesla transformer and graphite cathode (Supplementary Fig. 9a) were used to produce a single 20 ns SEB bunch, where the cross section (11 mm × 1.7 mm) of the SEB bunch was measured (Supplementary Fig. 9b). A single SEB bunch is injected through coupler 2 along the +*z* direction 0.5 mm above the surface of the CeSRR layer with an initial speed of 1.94 × 10^8^ m s^−1^. Due to space charge effects, the beam speed is slowed to 1.64 × 10^8^ m s^−1^ at the entrance of the LHM, meaning that the effective accelerating voltage decreases from 160 to 99.7 kV (Supplementary Fig. 10). To ensure the beam transport along the constructed structure, a solenoid is employed to produce an axial magnetic flux density (*B*_z_=1.06 T) (Supplementary Figs 11–13). A piece of graphite collector is used to collect the residual electrons from the SEB bunch (Supplementary Fig. 9c). The vacuum of the sealed system is maintained at the level of 3.8 × 10^−4^ Pa to ensure negligible beam-gas interactions. According to the synchronous condition and the space charge effects presented before, the SEB bunch with the initial speed of 1.64 × 10^8^ m s^−1^ can effectively interact with the TM-dominant mode in the LHM at a frequency slightly below 2.98 GHz. The power spectral densities of microwave signals at ports 2 and 1 are measured (Supplementary Fig. 14a) and shown in Fig. 4c. We see that the peak power at 2.847 GHz at port 1 is 17.6 dB lower than the 2.850 GHz RCR at port 2, where the signal frequency at port 1 is almost the same as that at port 2 (only a 3 MHz difference between the two frequency peaks, well within the margin for experimental error). In addition, we find that the experimental results (Fig. 4c) are in good agreement with the above simulated results (Fig. 4b). Therefore, these facts show that the RCR is indeed observed in the experiment.

## Discussion

We have established the left-handed behaviour of our CeSRR-loaded waveguide by using the S-parameter retrieval method and the Drude model to obtain a negative permittivity and permeability over the frequency range (2.83−3.05 GHz). We further confirmed the left-handed behaviour from the dispersion curves obtained from the HFSS simulation, and determined from the phase spectra. The origin of the negative permittivity results from the loaded metallic resonators (here the CeSRRs) and the negative permeability comes from the hollow waveguide operating below the cutoff frequency for the TM_11_ mode. In conclusion, the above methods indicate that our constructed structure can be considered as an LHM.

The Cherenkov radiation angle is formed by the direction of the electrons' movement (+*z* direction) and the direction of the Cherenkov radiation at a certain position, which is represented by the time-averaged Poynting vector. As an important parameter, it is investigated by using CST PIC simulations on the realistic structure model shown in Fig. 2a. The simulated results are illustrated in Fig. 4d. We find that the Cherenkov radiation angles are dependent on position and are greater than ∼150°. This fact confirms the anisotropy of the LHM and reveals the reversed behaviour of the Cherenkov radiation in the LHM from another viewpoint.

In addition, we investigate the origin of the small signal detected at port 1. In the RCR experiment, the microwave signal at port 1 is ∼5.5* *ns later than that at port 2, which is the signal propagation time from port 2 to port 1 due to an unavoidable reflection (Supplementary Fig. 14b and Supplementary Note 4). As stated before, the frequency of the signal at port 1 is the same as the RCR and the power is much smaller than that at port 2. These facts certify that the signal detected at port 1 is indeed the reflected one from the RCR signal at port 2, not another genuine signal excited by the SEB bunch.

In the meantime, we study the effect of the beam voltage on the RCR frequency, which is a crucial support for the negative band (corresponding to the backward dispersion), as shown in Fig. 1b. The simulated and measured results indicate that the RCR frequency increases with increasing beam voltage (Supplementary Fig. 15 and Supplementary Note 4). This finding is consistent with the prediction from the synchronous condition. This is because the slope of the beam line shown in Fig. 1b increases as the beam voltage increases, and the RCR frequency accordingly increases. Therefore, the RCR frequency can be tuned by changing the beam voltage.

This experiment has verified the one remaining theoretical prediction made by Veselago that RCR can be produced by moving charged particles in the LHMs. The dispersion curves and Cherenkov radiation angles reveal the reversed behaviour of Cherenkov radiation. By manipulating this artificial electromagnetic material, we can readily exploit this unusual RCR in a range of devices, such as next-generation high-power, miniaturized vacuum electronic devices in microwaves, millimeter waves, even terahertz waves^40^, ^41^, ^42^, ^43^, ^44^, particle detection in particle physics^15^,^16^, miniaturized accelerating cavity and bunch diagnostics in accelerator physics^45^, a device of metamaterial characterization on the macroscopic properties in material science^21^, and new types of plasmonic couplers in optics^29^.

## Methods

### Fabricated components

The constructed structure with the dimensions of 14.5 mm × 14.5 mm × 290 mm was built by the oxygen-free copper. The CeSRR layer with the dimensions of 14.5 mm × 1.2 mm × 290 mm was fabricated using the low-speed wire electrical discharge machining and the SW1 was fabricated by the wire electrical discharge machining (Supplementary Fig. 4a). Couplers 1 and 2 were also built by the oxygen-free copper (Supplementary Fig. 4b–e). The SW2 with a cross section of 60 mm × 60 mm is used to connect the SW1. The designed coaxial line (Supplementary Fig. 4f) is composed of an arc-type inner conductor with the radius of 0.6 mm, an outer conductor with the radius of 2.7 mm and the filled Teflon (the relative permittivity *ɛ*_r_=2.1 and loss tangent tan *δ*=0.0002). The cathode components were fabricated by the wire electrical discharge machining. The cross section of the graphite cathode is a rectangle with dimensions 12 mm × 2 mm. The solenoid consisting of a copper wire with a width of 6.4 mm and a thickness of 3.4 mm and a supporting stainless steel cylinder with a radius of 65 mm and a length of 632 mm were constructed (Supplementary Fig. 12). The isostatic pressing graphite collector with the dimensions of 55 mm × 55 mm × 20 mm was fabricated by the wire electrical discharge machining (Supplementary Fig. 9c).

### Experimental apparatus and setup

In the experiments introduced in the main text, an Agilent N5230A PNA-L Network Analyzer, an SG-42 Digital Panel Meter, a Tesla transformer, two kinds of attenuators (DTS200-50dB-4G and DTS30-10dB-4G), an Agilent DSO80804B Oscilloscope, an SHX-803-S-4 detector, an Agilent 83732B signal generator and an Agilent N1913A power meter were employed. The experimental setup used in the transmission experiments is illustrated in Supplementary Fig. 5. The Agilent N5230A PNA-L Network Analyzer is used to measure the S-parameters. The simplified block diagram for the RCR experiments is shown in Supplementary Fig. 8. To measure the RCR power, we used the SHX-803-S-4 detector. However, the detector was not included while measuring the RCR frequency (Supplementary Note 4).

### Data availability

The data that support the findings of this study are available from the corresponding authors upon reasonable request.

## Additional information

**How to cite this article:** Duan, Z. *et al*. Observation of the reversed Cherenkov radiation. *Nat. Commun.* **8,** 14901 doi: 10.1038/ncomms14901 (2017).

**Publisher's note**: Springer Nature remains neutral with regard to jurisdictional claims in published maps and institutional affiliations.

## Supplementary Material

Supplementary Information Supplementary Figures, Supplementary Notes and Supplementary References
