# Supplementary material for: Observation of the reversed Cherenkov radiation
Source: Nat Commun. 2017 Mar 23;8:14901. doi: 10.1038/ncomms14901 (PMC5376646; doi:10.1038/ncomms14901)
Supplement: Supplementary Information — Supplementary Figures, Supplementary Notes and Supplementary References [file ncomms14901-s1.pdf]

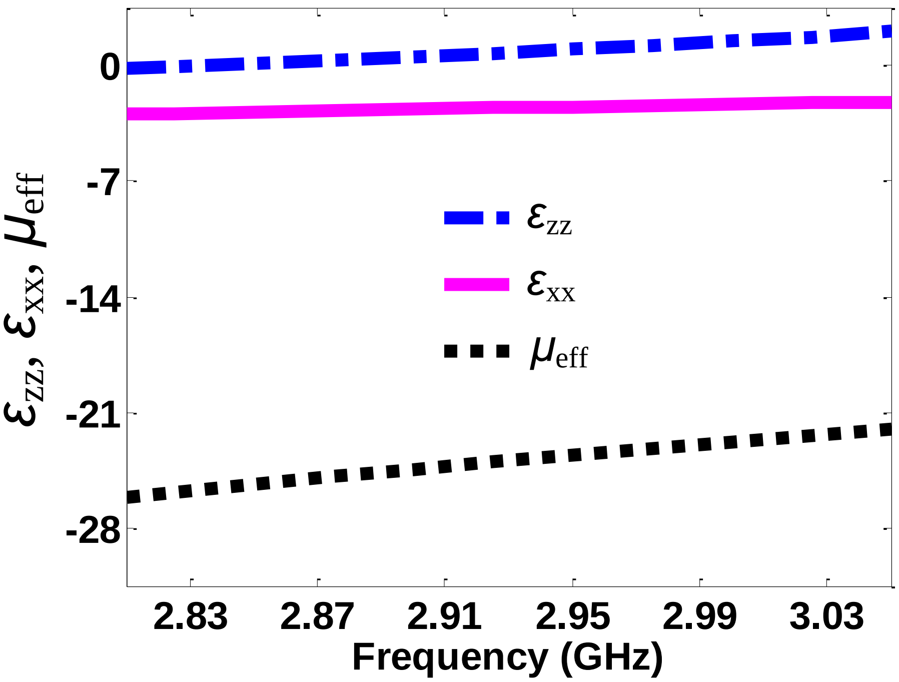

Supplementary Figure 1 | Effective constitutive parameters as a function of frequency. Both ε_{xx} and μ_{eff} are negative over the frequencies from 2.83 GHz to 3.05 GHz.

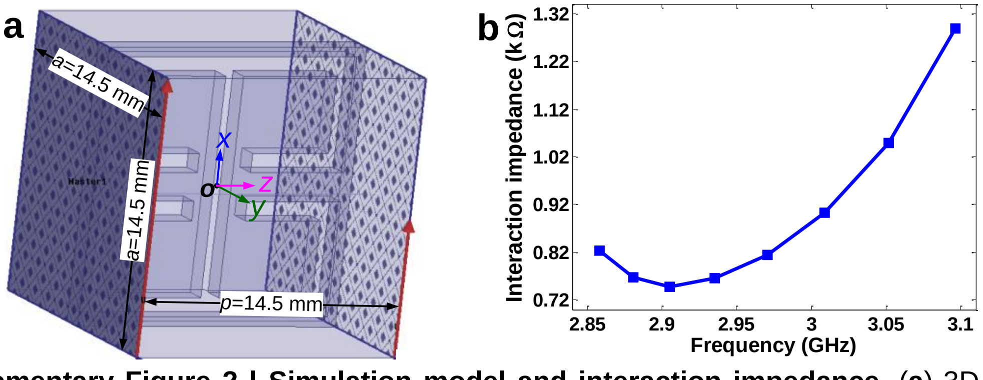

Supplementary Figure 2 | Simulation model and interaction impedance. (**a**) 3D HFSS simulation model for dispersion and interaction impedance. (**b**) Interaction impedance versus frequency. The interaction impedance is averaged at the cross section of the single sheet electron beam (SEB) bunch. The SEB bunch's bottom surface is 0.5 mm above the surface of the layer of complementary electric split ring resonators (CeSRRs) and its cross section is 12 mm×2 mm.

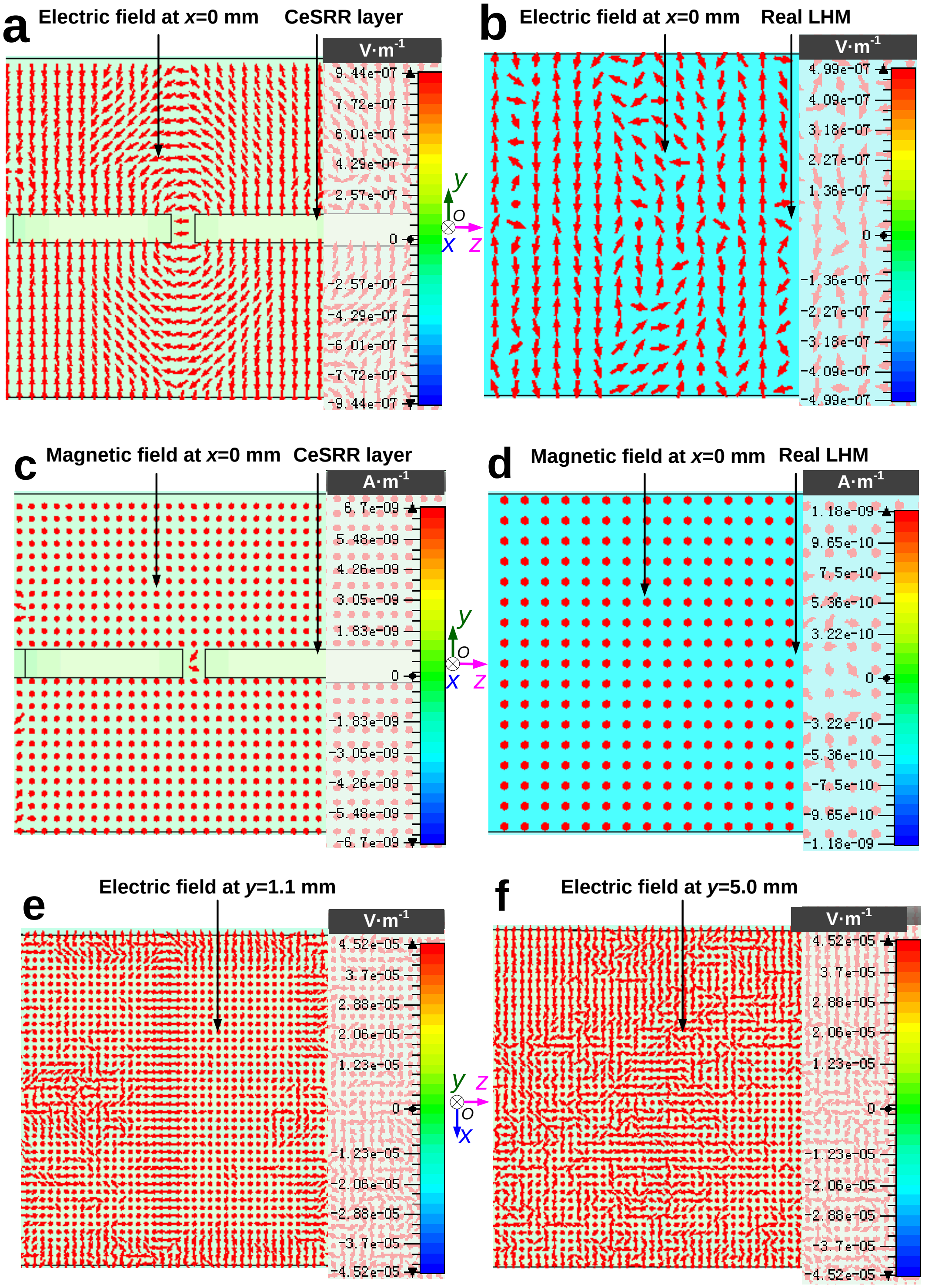

Supplementary Figure 3 | Field distributions of the TM–dominant mode at 2.85 GHz. (**a**) Electric field of *yz*–plane at *x*=0 in our constructed structure. (**b**) Electric field of *yz*–plane at *x*=0 in the real left–handed material (LHM). (**c**) Magnetic field of *yz*–plane at *x*=0 in our constructed structure. (**d**) Magnetic field of *yz*–pane at *x*=0 in the real LHM. (**e**) Electric field in *xz*–plane at y=1.1 mm in our constructed structure (close to the CeSRR layer). (**f**) Electric field in *xz*–plane at *y*=5.0 mm in our constructed structure (far away from the CeSRR layer).

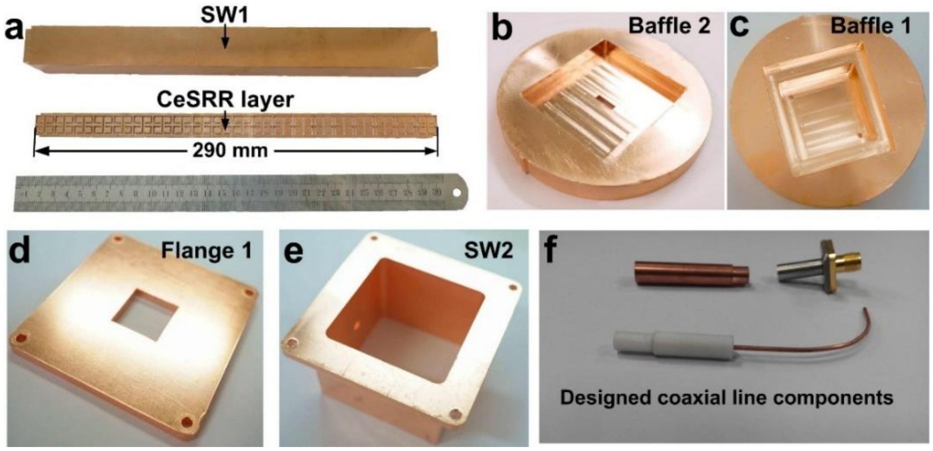

Supplementary Figure 4 | Photographs of fabricated components. (**a**) Constructed structure components. (**b**) Baffle 2. (**c**) Baffle 1. (**d**) Flange 1. (**e**) Another square waveguide (called SW2). (**f**) Designed coaxial line components. The constructed structure is made of the square waveguide (called SW1) and the CeSRR layer with 20 unit cells. Flange 1 is used for the SW1 and SW2. The designed coaxial line components are used to extract any generated signals.

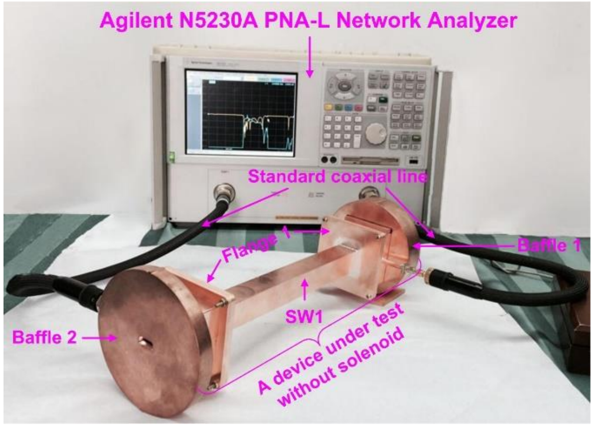

Supplementary Figure 5 | Photograph of the transmission experiment sets. An Agilent N5230A PNA–L Network Analyzer, two standard coaxial lines, and a device under test without solenoid are included.

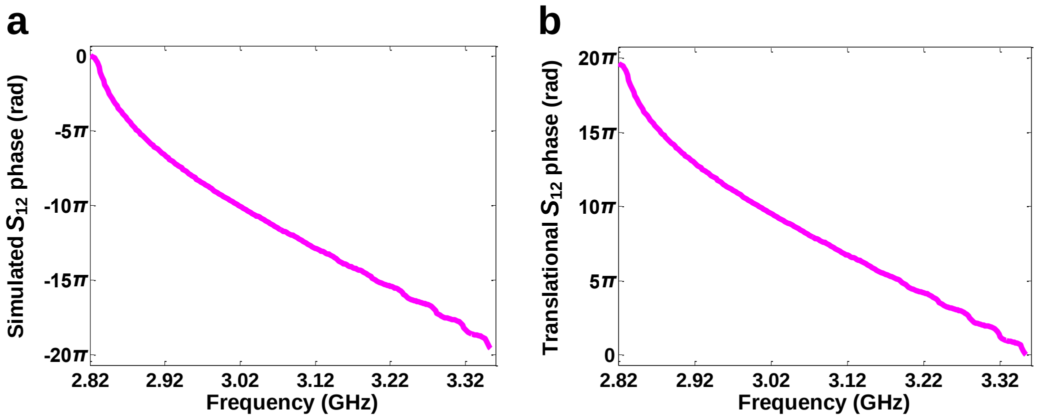

Supplementary Figure 6 | The phase spectra. (**a**) Simulated S_{12} phase versus frequency. (**b**) Translational S_{12} phase versus frequency.

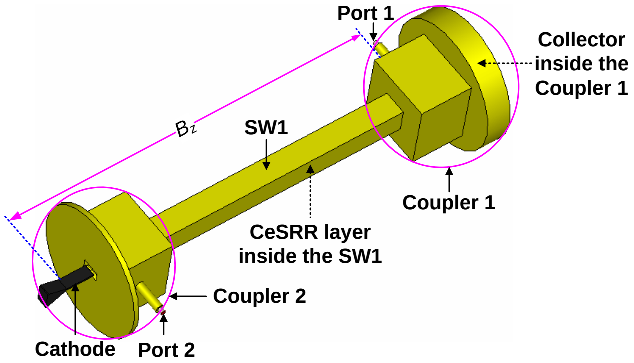

Supplementary Figure 7 | 3D CST PIC simulation model. The model is used to simulate the formation and amplification of the RCR in the constructed structure. The coordinate system is shown in Fig. 2b in the Main paper.

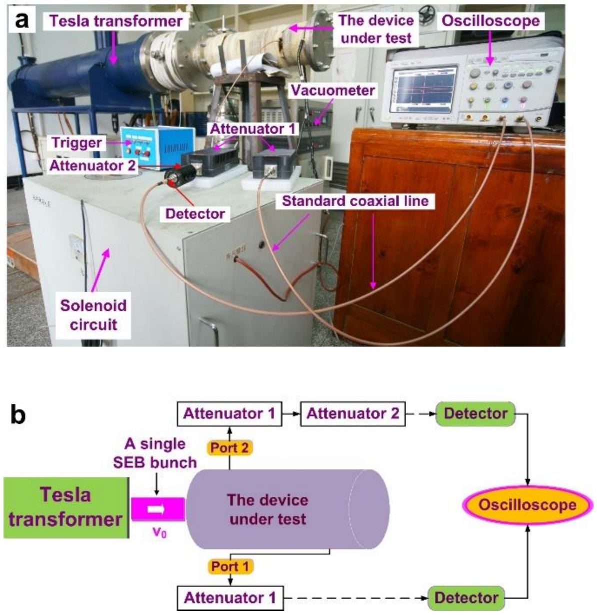

Supplementary Figure 8 | RCR experiments. (**a**) Photograph of the RCR experiment sets. (**b**) A simplified block diagram of the RCR experiment. Models of the experimental components in the diagram are listed here: DTS200–50dB–4G for attenuator 1, DTS30–10dB–4G for attenuator 2, SHX–803–S–4 for the detector, Agilent DSO80804B for the oscilloscope, and ZDF–5227 for the vacuometer.

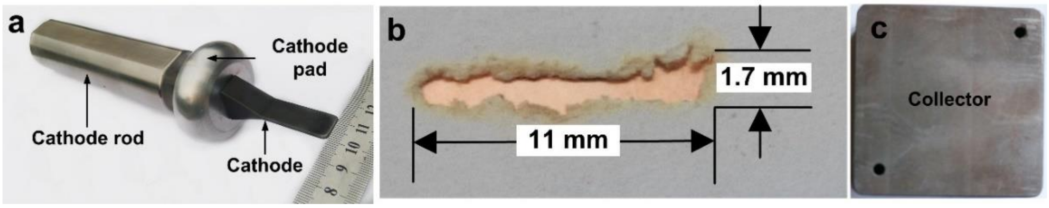

Supplementary Figure 9 | Photograph of the fabricated components. (**a**) Cathode components. (**b**) Measured cross section of the SEB bunch. (**c**) Isostatic pressing graphite collector. The cathode components consist of a cathode rod, a cathode pad, and a graphite cathode.

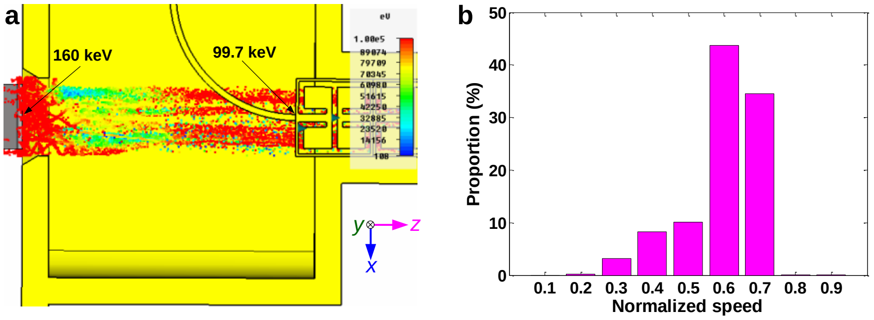

Supplementary Figure 10 | Electron trajectories and speed distributions. (**a**) Electron trajectories of the single SEB bunch. (**b**) Electron speed distributions at the entrance of the constructed structure. The electron speeds of the single SEB bunch are normalized to the speed of light in vacuum.

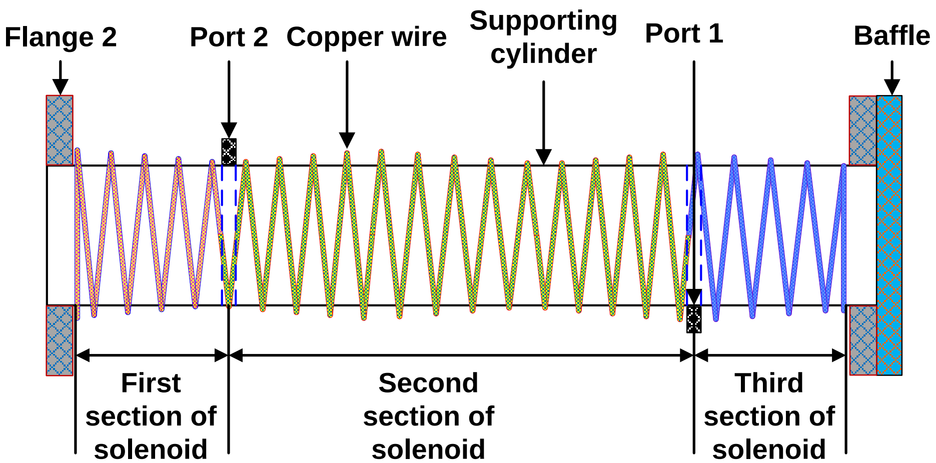

Supplementary Figure 11 | A sketch map of the solenoid. It is assumed to separate into three sections for calculation.

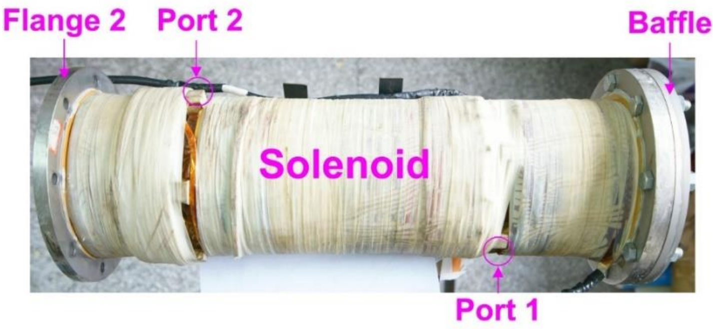

Supplementary Figure 12 | Photograph of the solenoid sample. It can generate the axial magnetic field for beam transport. Flange 2 is used to connect the Tesla transformer and the solenoid.

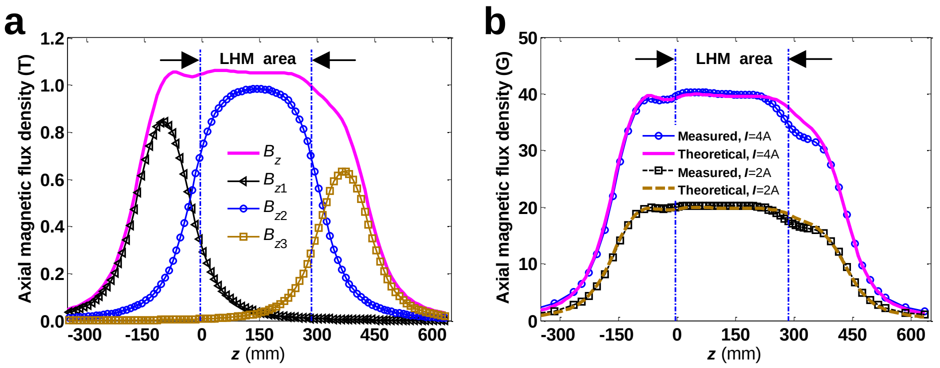

Supplementary Figure 13 | Axial magnetic flux density versus axial position. (**a**) Theoretical results for the current I=1.09 kA. (**b**) Comparisons between the theoretical and measured results for the direct currents I=2 A and 4 A.

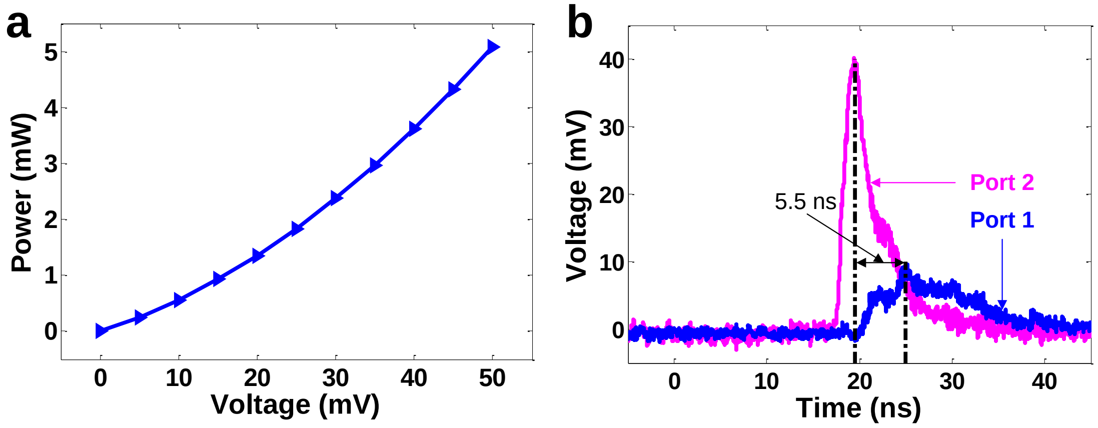

Supplementary Figure 14 | Measured results. (**a**) The corresponding relation between voltage and power for calibration. (**b**) Measured voltages for RCR at port 2 and its reflected one at port 1 versus time.

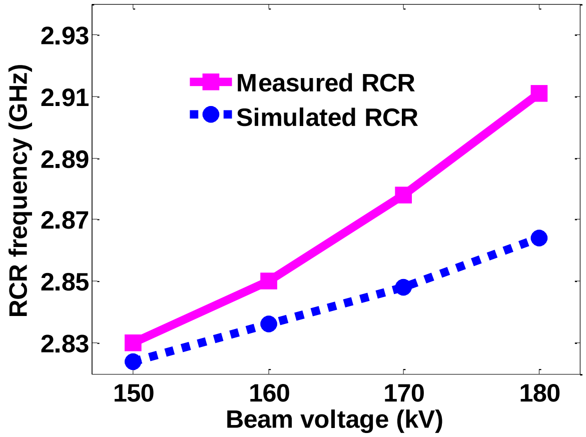

Supplementary Figure 15 | RCR frequency as a function of beam voltage. The RCR frequency increases with the increase of beam voltage.

Supplementary Note 1: Effective Constitutive Parameters of the LHM

For the constructed structure shown in Fig. 1a in the Main paper, the size of the unit cell of the complementary electric split ring resonator (CeSRR) and the size of the cross section for the square waveguide (14.5 mm ×14.5 mm, called SW1) are indeed much smaller than the free–space wavelength λ (the size is in the order of ~λ/7). Note that the period of CeSRRs is p=14.5 mm. The facts indicate that the constructed structure can be considered as the effective media. Hence, we use the S–parameter retrieval method[1] based on the HFSS[2] simulation for one CeSRR unit cell to obtain the effective permittivity tensor, which is written as

$$\bar{\bar{\varepsilon}} = \varepsilon_0 \bar{\bar{\varepsilon}}_{\text{eff}} = \varepsilon_0 \begin{bmatrix} \varepsilon_{\text{xx}} & 0 & 0 \\ 0 & 1 & 0 \\ 0 & 0 & \varepsilon_{\text{zz}} \end{bmatrix}, \tag{1}$$

where ε_0 is the permittivity in vacuum, and ε_{xx} and ε_{zz} are the effective permittivities in the x and z directions, respectively. The related details are presented in Supplementary Ref. 3.

Meanwhile, a hollow metallic waveguide operating in the microwave region can be regarded as a one–dimensional lossless magnetic plasma with respect to the electromagnetic wave propagation (here TM_{11} mode) along the axial direction[4–8]. Thus, the effective permeability is predicted by the Drude model

$$\mu = \mu_0 \mu_{\text{eff}} = \mu_0 \left(1 - \frac{\omega_{\text{c}}^2}{\omega^2} \right), \tag{2}$$

where μ_0 and μ_{eff} are the permeability in vacuum and the relative permeability, respectively, $\omega_{\text{c}} = 2\pi \text{f}_{\text{c}}$ is the plasma angular frequency and $\omega = 2\pi f$ is the angular frequency, f_c and f are the cutoff frequency and the operating frequency for the TM_{11} mode, respectively. Here f_c is given by[9]

$$f_c = \frac{1}{a\sqrt{2\varepsilon_0 \mu_0}}, \tag{3}$$

where a is the side length of the cross section of the hollow square waveguide.

As a consequence, the effective constitutive parameters are shown in Supplementary Fig. 1. It shows that in the frequency band (2.83 GHz–3.05 GHz), the effective media exhibit both the negative permittivity (ε_{xx}) and negative permeability (μ_{eff}). These facts confirm that the effective media presented here can be considered as a left–handed material (LHM).

We should mention that traditional slow–wave structure (SWS), such as a corrugated SWS in backward–wave oscillator (BWO), has larger size (the size is in the order of ~λ/3–~λ)[10–13] compared with our constructed structure (~λ/7). For a typical example, the unit cell dimension in a realized LHM[14] was roughly one–sixth the free space wavelength (~λ/6). Hence, the traditional SWS cannot be regarded as an effective medium. Even if we retrieve the effective constitutive parameters using the S–parameter retrieval method, the negative values cannot be found. These facts indicate that there is the principal difference between the constructed structure and the traditional SWS. This difference leads to the important advantages of our constructed structure relative to the traditional SWS, such as miniaturization and high interaction impedance shown below.

Supplementary Note 2: HFSS Simulations of Dispersion and Interaction Impedance

The constructed structure used in the experiments consists of a CeSRR layer and the SW1. As a novel SWS, its high–frequency characteristics include the dispersion relation and interaction impedance. The dispersion relation is the dependence of the phase velocity of electromagnetic waves on frequency. The interaction impedance K_c is used to characterize the degree of the interaction between the electron beam and excited electromagnetic waves (here RCR) and is written as[15]

$$K_c = \frac{|E_{z0}|^2}{2\beta_0^2 P}, \tag{4}$$

where $|E_{z0}|^2$ is amplitude of the longitudinal electric field of the n=0 space harmonic of the fundamental mode (n is the space harmonic index), β_0 is the phase constant of the n=0 space harmonic, and P is the power flow along the axial direction in the constructed structure.

Here we used the eigenmode solver and post processing in HFSS[2] to calculate the high–frequency characteristics of the constructed structure. The simulated model is shown in Supplementary Fig. 2a. In the simulation setup, the boundary conditions in the x and y directions are perfect electric boundary conditions, while those in the z direction are master and slave boundary conditions, and the tetrahedral mesh type is chosen. As a result, the simulated dispersion curve is shown in Fig. 1b in the Main paper and the interaction impedance as a function of frequency is plotted in Supplementary Fig. 2b. It can be seen that for the constructed structure the n=0 space harmonic of the fundamental mode exhibits the left–handed behavior or backward dispersion and its averaged interaction impedance is greater than ~750 Ω. This is because the large improvement of

the longitudinal electric field intensity leads to a dramatic increase of the interaction impedance. It should be noted that the interaction impedances of the commonly used helix and coupled−cavity SWSs at S band are about 100 Ω−200 Ω and about 300 Ω−400 Ω, respectively. Therefore, it is an obvious advantage for our constructed structure.

According to the physical mechanism of the Cherenkov−based vacuum electronic devices, such as traveling−wave tubes or BWOs[16], the higher the interaction impedance, the higher the electronic efficiency and the higher the output power. This is because the output power is approximately proportional to the 1/3−power of the interaction impedance. Therefore, the large improvement of the interaction impedance is helpful for increasing the electronic efficiency and the output power. This remarkable feature shows an important application of the RCR to vacuum electronic devices.

Supplementary Note 3: Transmission Simulations and Experiments

(1) Transmission simulations

In order to ensure the good transmission characteristics of electromagnetic waves in the constructed structure without any SEB bunch, we need two suitable couplers to input and output the signals (corresponding to couplers 2 and 1, respectively). Hence, we used the frequency domain solver in CST Microwave Studio[17] to design the two couplers. In the simulation setup, the boundary conditions in x, y, and z directions for the constructed structure except for the two port surfaces are perfect electric boundary conditions, and the tetrahedral mesh type is used. The two couplers presented here are confirmed by the amplitudes of the S−parameters from the full wave simulations, which are shown in Fig. 3a in the Main paper. As we can see, the two couplers have large transmission and small reflection coefficients. Therefore, the RCR signal excited by the single SEB bunch can be detected via the two well−designed couplers.

(2) Fabricated components

In order to carry out the transmission experiment, we fabricated the related experimental components. The constructed structure is shown in Supplementary Fig. 4a. For efficiently detecting the RCR signal from the constructed structure, we designed and built the two couplers, whose components are shown in Supplementary Fig. 4b−f. The two couplers are almost the same except that baffle 2 has a rectangular hole for letting the SEB bunch pass and baffle 1 has a deep groove for locating the collector.

(3) Transmission experiments

The platform of the transmission experiments is shown in Supplementary Fig. 5. In the experiment, we followed the procedures to measure the S−parameters using an Agilent N5230A PNA−L Network Analyzer. We first calibrated the Network Analyzer to ensure that the experimental results are accurate. Next, a device under test without solenoid is connected to the Network Analyzer with the two standard coaxial lines and then the S−parameters are measured. Finally, the experimental data of the S−parameters are exported from the Network Analyzer, as shown in Fig. 3b,d in the Main paper.

We compare the amplitudes of the simulated S−parameters (Fig. 3a in the Main paper) with those of the measured S−parameters (Fig. 3b in the Main paper), and find that the measured and simulated results agree well with each other. These facts confirm that the constructed structure with the two couplers is suitable for observing the RCR in the experiment.

In addition, the left−handed behaviors can be also verified by examining the phase spectra. The phases of the simulated and measured S_{12} parameters are shown Fig. 3c,d, respectively in the Main paper. Here we converted the S_{12} phases to the dispersion relation by the following way. First, we obtained the alternative S_{12} phases versus frequency using the frequency domain solver in CST Microwave Studio[17], as shown in Supplementary Fig. 6a. Secondly, we made a translation to form Supplementary Fig. 6b. Finally, the phase advance ϕ for a single unit cell is obtained by the total phase shown in Supplementary Fig. 6b divided by the number of CeSRRs (here 20). Thus, the dispersion curves from the simulated and measured S_{12} phases are shown in Fig. 3e in the Main paper. It can be seen that the two dispersion curves are in good agreement with the HFSS simulation results. The fact further verifies the left−handed behavior or backward dispersion of our constructed structure.

Supplementary Note 4: RCR Simulations and Experiments

(1) RCR simulations

After the transmission simulations, we used the particle−in−cell (PIC) solver in CST Particle Studio[17] to study the RCR before performing the RCR experiments. The 3D simulation model, as shown in Supplementary Fig. 7, consists of a graphite cathode, a constructed structure (the CeSRR layer and the SW1), coupler 1, coupler 2, the axial magnetic flux density (B_z=1.06 T), and the collector inside the coupler 1. In the simulation setup, the boundary conditions in x, y, and z directions are perfect electric boundary conditions, and the hexahedral mesh type is chosen. To ensure enough

simulation accuracy, the local mesh refinement is employed to increase the mesh cell numbers in the SEB bunch and constructed structure area. Here we set up the PIC emission model (DC model) and the simulation time (20 ns) in CST Particle Studio[17] to produce a single 160 kV, 1.55 kA, 20 ns pulse SEB bunch with the cross section of 12 mm×2 mm, which passes 0.5 mm above the surface of the CeSRR layer. Thus, we obtained the normalized power flow along the z–axis, and the power spectral densities of signals at ports 2 and 1, the Cherenkov radiation angles at different positions as shown in Fig. 4a,b,d in the Main paper, respectively. The results state that the power of the 2.829 GHz signal at port 1 is 16.9 dB lower than that of the 2.836 GHz one at port 2, and the slight frequency difference between the two signals (7 MHz) is well within the computational accuracy. This fact shows the signal at port 2 is the RCR and the one at port 1 is the signal reflected from port 2.

In order to confirm the TM–dominant mode, we used the above PIC simulations on the constructed structure to obtain the field distributions. The electric and magnetic fields in yz–plane at x=0 mm are shown in Supplementary Fig. 3a,c, respectively, and the electric fields in xz–plane at y=1.1 mm and y=5.0 mm are shown in Supplementary Fig. 3e,f, respectively. It can be found that the constructed structure can support a TM–dominant mode. This is because the z–component of electric field is finite, while the z–component of magnetic field is close to zero. Meanwhile, we used the time domain solver in CST Microwave Studio[17] to simulate the distributions of electric and magnetic fields along the z–axis in the real LHM. In the simulation setup, the effective parameters shown in Supplementary Fig. 1 are used for describing the real LHM. From Supplementary Fig. 3b,d, we find that the field distributions in the real LHM are similar to those shown in Supplementary Fig. 3a,c. This is a great supporting argument for the effective media presented here.

As shown in Fig. 4b,c in the Main paper, the peak frequency of RCR is lower than the frequency corresponding to 160 kV (SEB line 1), as shown in Fig. 1b in the Main paper. This is because the kinetic energy of the SEB bunch at the entrance of the LHM would be lower than that at the surface of the cathode due to space charge effects[18]. In order to obtain the actual initial speed of the SEB bunch that begins to interact with the excited RCR, we used the PIC solver in CST Particle Studio[17] to obtain the electron trajectories and speed distributions at the entrance of the constructed structure, as illustrated in Supplementary Fig. 10a,b, respectively. The averaged velocity $\langle \mathbf{v}_0 \rangle$ of the SEB bunch can be calculated by

$$\langle \mathbf{v}_0 \rangle = \frac{1}{N}\sum_{n=1}^{N} v_n \, \mathbf{z} \,, \tag{5}$$

where N is the total number of electron at the entrance, v_n is the speed of the nth electron, and $\mathbf{z}$ is the unit vector of z–axis. As a result, the averaged speed is 1.6410×10^8 m s^{-1}, which corresponds to an averaged kinetic energy of 99.7 keV (SEB line 2), as shown in Fig. 1b in the Main paper.

(2) Solenoid design, fabrication, and measurement

To implement the pulse magnetic field for beam transport, we designed a solenoid to produce a 1.09 kA, 16 ms pulse current. The solenoid is comprised of a supporting cylinder with flange 2 and a spirally enwinding copper wire on the outer surface of the supporting cylinder, as shown in Supplementary Fig. 11.

The copper wire has a rectangular cross section with the width of w_1=6.4 mm and the thickness of t_1=3.4 mm. One end of the supporting cylinder is linked to the Tesla transformer and the other end is sealed by the baffle to maintain the vacuum of the system. The solenoid is assumed to separate into three sections (j=1, 2, 3) for calculation. For each section of the solenoid, the magnetic flux density along the central axis of the solenoid can be expressed as

$$B_{zj} = \frac{\mu_0 n_1 n_2 I}{2}\left[\left(\frac{L_{sj}}{2}+z_j-z_{0j}\right)\ln\frac{r_{oj}+\sqrt{r_{oj}^2+\left(\frac{L_{sj}}{2}+z_j-z_{0j}\right)^2}}{r_{ij}+\sqrt{r_{ij}^2+\left(\frac{L_{sj}}{2}+z_j-z_{0j}\right)^2}}+\left(\frac{L_{sj}}{2}-z_j+z_{0j}\right)\ln\frac{r_{oj}+\sqrt{r_{oj}^2+\left(\frac{L_{sj}}{2}-z_j+z_{0j}\right)^2}}{r_{ij}+\sqrt{r_{ij}^2+\left(\frac{L_{sj}}{2}-z_j+z_{0j}\right)^2}}\right], \tag{6}$$

where I is the exciting current of the solenoid, $n_1 = 1/w_1$ and $n_2 = 1/t_1$ are the turn number in unit length and layer number in unit thickness, respectively, L_{sj} is the length of each section of the solenoid, r_{ij} and r_{oj} are the inner and outer radii of each section of the solenoid, respectively, and z_{0j} and z_j are the axial positions of midpoint and measured point at each section of the solenoid axis, respectively[19,20]. Therefore, the total axial magnetic flux density B_z can be written as B_z=B_{z1}+B_{z2}+B_{z3}.

Based on the above design, we built a solenoid sample, as shown in Supplementary Fig. 12. According to equation (6), the axial magnetic flux density B_z with maximum 1.06 T is plotted in Supplementary Fig. 13a. To verify the theoretical calculation, the axial magnetic flux density is measured for small direct current cases. The reasons are listed here: 1) it is hard to directly measure the axial magnetic flux density at a given time due to the time–varying characteristic of the pulse current; 2) the big direct current source

with kA order of magnitude is not available in our laboratory; 3) because the pulse length of the single SEB bunch (20 ns) is much smaller than the period of the pulse current (16 ms), the pulse current around the 1.09 kA can be considered as a direct current during the time of 20 ns; 4) it is feasible to measure the axial magnetic flux density in small direct current cases to verify the theoretical prediction, since the axial magnetic flux density is proportional to the current predicted by equation (6).

In this experiment, we utilized the SG−42 Digital Panel Meter (DPM) to measure the axial magnetic flux density of the solenoid when the direct currents are 2 A and 4 A, respectively. We first fixed the probe of DPM on a long ruler using rubber belts. The end of probe is aligned with the zero graduation line of the ruler, and the probe is parallel to the ruler. Then we placed the probe and the long ruler in the inner middle of solenoid and changed the z−position of the probe every 10 mm from −350 mm to 650 mm to obtain a series of magnetic flux density data. As a consequence, when the direct currents are 2 A and 4 A, respectively, the theoretical and measured magnetic flux densities as a function of z are plotted in Supplementary Fig. 13b, showing that the theoretical and measured results agree well.

(3) RCR experiments

Before the RCR experiments, we built the cathode components, as shown in Supplementary Fig. 9a. We fixed a white paper on the surface of baffle 2 to measure the cross section of the SEB bunch. The measured dimensions of the cross section is 11 mm×1.7 mm (Supplementary Fig. 9b), which is close to the nominal value 12 mm×2 mm. The fact verifies that the SEB is indeed produced. In addition, the collector is used to collect the residual electrons (Supplementary Fig. 9c).

The platform photograph of the RCR experiment and the corresponding block diagram are shown in Supplementary Fig. 8a,b, respectively. When the SEB bunch is generated by the cathode and the Tesla transformer, it fast moves through the constructed structure under the focusing of the axial magnetic field. Thus, it can effectively interact with the excited RCR, and then the RCR signal is amplified. As a result, the two signals including the RCR and its reflected signals can be exported from ports 2 and 1, respectively, attenuated by attenuators 1 and 2, and finally received by the oscilloscope.

Meanwhile, we calibrated the detector at 2.85 GHz, which is the RCR frequency, using an Agilent 83732B signal generator, an Agilent N1913A power meter, and an Agilent DSO80804B oscilloscope. To obtain the corresponding relation between voltage and power for calibration, we used a power meter to measure the power of a signal directly

generated from the signal generator. Meanwhile, the signal is converted to a voltage by the detector and read out from the oscilloscope (Supplementary Fig. 14a). After the calibration, the power of the two microwave signals at ports 2 and 1 are attenuated by 63 dB and 53 dB, respectively, and then are converted to the voltages by the two detectors, and finally read out from the oscilloscope. According to the corresponding power–voltage relation, the power of the RCR and its reflected signals at ports 2 and 1 is obtained. In addition, as shown in Supplementary Fig. 14b, the delayed 5.5 ns of the signal at port 1 relative to that at port 2 is the propagation time for the RCR signal reflected from port 2 to port 1.

Additionally, in order to obtain the normalized power spectral densities of the two signals, another measurement is performed under the same condition without the detector. We used the oscilloscope to extract the signals versus time and obtained the normalized power spectral densities using Fourier transform, which are shown in Fig. 4c in the Main paper. Based on these facts, we conclude that the RCR signal is indeed observed and the much weaker signal at port 1 is the reflected RCR signal, not a genuine signal directly produced in the LHM.

At last, we investigate the effect of the beam voltage on the RCR frequency using simulations and experiments. The RCR frequency as a function of beam voltage is shown in Supplementary Fig. 15. We see that the RCR frequency increases with the increase of beam voltage. The finding is in step with the synchronous condition[21] (shown in Fig. 1b in the Main paper).

Supplementary References

1. Smith, D. R. Analytic expressions for the constitutive parameters of magnetoelectric metamaterials. *Phys. Rev. E* **81**, 036605 (2010).
2. HFSS software, Copyright Ansoft. *Pittsburgh, USA.*
3. Duan, Z. Y., Hummelt, J. S., Shapiro, M. A. & Temkin, R. J. Sub–wavelength waveguide loaded by a complementary electric metamaterial for vacuum electron devices. *Phys. Plasmas* **21**, 103301 (2014).
4. Marqués, R., Martel, J., Mesa, F. & Medina, F. Left–handed–media simulation and transmission of EM waves in subwavelength split–ring–resonator–loaded metallic waveguides. *Phys. Rev. Lett.* **89**, 183901 (2002).
5. Kondrat'ev, I. G. & Smirnov, A. I. Comment on ''Left–handed–media simulation and transmission of EM waves in subwavelength split–ring–resonator–loaded metallic waveguides''. *Phys. Rev. Lett.* **91**, 249401 (2003).
6. Hrabar, S., Bartolic, J. & Sipus, Z. Waveguide miniaturization using uniaxial

negative permeability metamaterial. *IEEE Trans. Antennas and Propag.* **53**, 110–119 (2005).

7. Esteban J., Camacho–Peñalosa C., Page J. E., Martín–Guerrero T. M. & Márquez–Segura E. Simulation of negative permittivity and negative permeability by means of evanescent waveguide modes—theory and experiment. *IEEE Trans. Microw. Theory Tech.* **53**, 1506–1514 (2005).
8. Xu, H., *et al*. Effective–medium models and experiments for extraordinary transmission in metamaterial–loaded waveguides. *Appl. Phys. Lett.* **92**, 041122 (2008).
9. Pozar, D. M. *Microwave Engineering* (John Wiley & Sons, Inc., Hoboken, 2012).
10. Mineo, M. & Paoloni, C. Double–corrugated rectangular waveguide slow–wave structure for terahertz vacuum devices. *IEEE Trans. Electron Devices* **57**, 3169–3175 (2010).
11. Amin, Md. R. & Ogura, K. Temporal growth study in trapezoidally corrugated slow–wave structure for backward–wave oscillator. *IEEE Trans. Plasma Sci.* **41**, 2257–2263 (2013).
12. Totmeninov, E. M. L–band relativistic traveling wave oscillator based on a circular corrugated waveguide. *IEEE Trans. Plasma Sci.* **44**, 1276–1279 (2016).
13. Kato, M., *et al*. Development of 100 GHz interdigital backward–wave oscillator. *36th Consultative Meeting of INIS Liaison Officers*, Vienna, Austria (2012).
14. Shelby, R. A., Smith, D. R. & Schultz, S. Experimental verification of a negative index of refraction. *Science* **292**, 77–79 (2001).
15. Chermin, D., Antonsen, T. M. Jr. & Levush, B. Exact treatment of the dispersion and beam interaction impedance of a thin tape helix surrounded by a radially stratified dielectric. *IEEE Trans. Electron Devices* **46**, 1472–1483 (1999).
16. Johnson, H. R. Backward–wave oscillator. *Proc. IRE* **43**, 684–697 (1955).
17. CST software, Copyright CST. *Darmstadt, Germany*.
18. Pierce, J. R. Rectilinear electron flow in beams. *J. Appl. Phys.* **11**, 548–554 (1940).
19. Kroon, D. J. *Electromagnets* (Boston Technical Publishers Inc., Boston, 1968).
20. Knoepfel, H. *Pulsed High Magnetic Field* (North–Holland Publishing Co., Amsterdam, 1970).
21. Rowe, J. E. *Nonlinear Electron–wave Interaction Phenomena* (Academic press, New York, 1965).
